# National Emergency Bariatric Surgical Audit (NEBSA): a protocol for a multi-center prospective study of unplanned interventions following emergency bariatric surgery

**DOI:** 10.1097/SP9.0000000000000037

**Published:** 2025-03-20

**Authors:** Fahad M. Iqbal, Alan Askari, Matthew J. Lee, Marianne Hollyman, Roxanna Zakeri, Dimitri J. Pournaras, Omar Al-Taan, Aruna Munasinghe, Aly Mohamed

**Affiliations:** a Department of Surgery and Cancer, Imperial College London, St Mary’s Hospital, London, United Kingdom; b Department of General Surgery, Luton & Dunstable Hospital, Luton, United Kingdom; c Division of Clinical Medicine, Faculty of Medicine, Dentistry & Health, University of Sheffield, Sheffield, United Kingdom; d Department of Bariatric Surgery, Musgrove Park Hospital, Taunton, United Kingdom; e Department of General Surgery, University College London Hospital NHS Foundation Trust, London, United Kingdom; f Department of Bariatric and Metabolic Surgery, Southmead Hospital, North Bristol NHS Trust, Bristol, United Kingdom; g Department of General Surgery, Hillingdon Hospital, London, United Kingdom

**Keywords:** bariatric surgery, emergency medical services, health care surveys, outcome and process assessment (health care), surgical complications

## Abstract

**Introduction:**

The advent of bariatric surgery as a widespread intervention is paralleled by comprehensive data capture in bariatric registries following elective surgery. However, significant challenges hinder tracking the incidence and nature of severe complications in the context of bariatric surgery. As the prevalence of bariatric procedures escalates, the establishment of a dedicated, prospective complication registry becomes imperative. Such an initiative would facilitate a nuanced understanding of bariatric surgical emergency (BSE) within the current healthcare milieu, enhance economic evaluations, elucidate long-term patient outcomes, and inform requisite adjustments in professional training. This study is designed to capture and assess the ramifications of emergency bariatric surgical practices within the United Kingdom.

**Methods and analysis:**

We propose a prospective, multi-center, audit of emergency bariatric surgical activity in all UK hospitals. Eligible participants are those who undergo any intervention or procedure (surgical or endoscopic) to diagnose or treat BSE. Primary outcome measures will include hospital length of stay, rates of complications (Clavien–Dindo), and 30-D mortality. Secondary outcomes will assess the broader impacts and patterns of care, including variations in practice and resource utilization across the nation, rates of outpatient follow-up, and the frequency of subsequent procedures (surgical or endoscopic) post-BSE. Additionally, the study will investigate potential predictors for patients’ choice between state-funded and self-pay bariatric surgery options, considering factors such as ethnicity and previous engagement with NHS-specialized weight loss pathways.

**Ethics and dissemination:**

This study will be registered as clinical audit at each participating hospital. The protocol will be disseminated through the British Obesity and Metabolic Surgery Society network and using a targeted social media-based strategy in the UK.

## Introduction

Although bariatric surgery is considered safe, exhibiting a mortality rate between 0.03 and 0.2%, the rate of early complications and readmissions varies across different bariatric procedures, typically affecting fewer than 6% of participants^[1]^. Emergency admissions following bariatric surgery often necessitate nutritional support or due to surgical complications which may manifest in either the early or late postoperative periods^[2]^. These participants may present at non-specialist hospitals or at institutions other than their initial surgical center, influenced by factors such as insurance coverage or geographical proximity^[3]^. In the United Kingdom, standard protocol dictates referral to specialized bariatric centers in the event of complications^[4]^. Despite potential increases in travel distance and time, treatment at such specialized centers has been correlated with enhanced patient outcomes^[5]^.

**Strengths and limitations of this study**

- NEBSA determines the interventions and complication rate of bariatric surgery.
- NEBSA describes patient demographics and engagement with NHS weight management processes.
- NEBSA investigates the need for nutritional support in the postoperative bariatric population across the UK.
- As an observational study, NEBSA findings are indicative of association rather than definitive causality.
- NEBSA is a starting point for further inquiry and will aid the design of future studies.

Over the past four years, the United Kingdom has averaged approximately 6,000 bariatric procedures annually. A notable decline in these numbers in 2020 can be attributed to the COVID-19 pandemic; however, recovery in surgical activities was observed in 2021^[6]^. Prior to the pandemic, the National Health Service (NHS) funded 75% of bariatric surgeries, a proportion that has remained relatively constant^[7]^. The enduring impact of the pandemic on the full resurgence of NHS-funded bariatric procedures, and the potential shift of participants toward the independent sector, remains an area of ongoing observation.

The current data reported to the National Bariatric Surgery Registry (NBSR) are a comprehensive dataset describing the patient population undergoing elective bariatric surgery and have formed a substantial portion of the eighth International Federation for the Surgery of Obesity and Metabolic Disorders (IFSO) registry report^[8]^. Nonetheless, there is a recognized knowledge gap concerning the patterns of presentation and impact of complications following bariatric surgery. There exists a discrepancy between the overall complication rate recorded in the Hospital Episode Statistics register and that reported to the NBSR, identified as 2.38%. This disparity may stem from participants presenting to alternate hospitals other than the index hospital where their surgery was performed, and efforts are being made to rectify these discrepancies^[7]^. Equally, some bariatric centers and most non-bariatric hospitals may not subscribe to NBSR, further widening this knowledge gap. This presents an opportunity for an all-inclusive database to include all centers admitting and treating patients with complications following bariatric surgery.

Barriers and facilitators of the phenomenon of bariatric medical tourism have been identified^[9]^. However, this phenomenon, along with self-funded bariatric surgery, and its impact on the NHS’s emergency surgery services have not been extensively explored^[10]^. Analysis of the current provision of bariatric surgery in the UK suggests a drastic investment is required in both NHS and private sectors to meet the predicted demand and highlights the high cost in treating bariatric complications^[11]^. To date, only a single UK study has described emergency surgical activities in a bariatric center, covering the period from 2011 to 2013 – a time when the provision of bariatric surgery by the NHS, as well as by independent sectors within and outside the UK, was markedly different from current practices^[12]^.

Consequently, this study aims to ascertain contemporary trends and outcomes, such as hospital length of stay, mortality, and severity of complications, in the management of emergency admissions following bariatric surgery across the UK through prospective and continuous data collection. By obtaining a substantial overview of current management of bariatric complications, this study intends to provide insights into service delivery, associated costs, and training needs, thereby informing future policy development.

## Methods and analysis

### Overall design

This is a prospective, multi-center, cross-sectional study of emergency bariatric surgical activity in the UK. Participants will be identified at admission as per the eligibility criteria, and their outcomes will be prospectively recorded. Data will be collected online via a bespoke data collection tool. Dissemination through the British Obesity and Metabolic Surgery Society (BOMSS) committee and clinicians at registered bariatric centers and acute non-bariatric centers in the UK will be undertaken. This protocol was developed in accordance with recommendations from Standards for QUality Improvement Reporting Excellence (SQUIRE) guidelines^[13]^.

### Study setting

All hospitals in the UK offering emergency gastrointestinal surgery and bariatric centres will be invited to participate. Participants will be identified over a 6-month period. This period length ensures compliance of hospitals participating in the audit with data collection and validation. This study duration also aims to maximize resident and allied healthcare professional participation, who will be involved in data collection, a model that has been shown to be successful elsewhere^[14,15]^. The data collection period will be planned between rotation dates. Provisionally, the initial study period will be from 9 October 2024 to 9 April 2025. However, other hospitals have registered later than the planned start date, and data collection is still ongoing for a separate 6-month cycle. At the time of submitting the protocol manuscript (December 2024), some centers are still contributing to the first round of data collection. A second period of data collection is scheduled from June to December 2025.

### Inclusion criteria

- Admissions from i) the emergency department, clinic, urgent care, or primary care to the bariatric surgical team as an emergency; ii) from an inpatient team as an urgent referral; iii) a return to theater following an elective bariatric procedure; and iv) another hospital via patient transfer to a specialized unit.
- Participants undergoing any intervention or procedure to treat or diagnose bariatric complications, (e.g. OGDs, interventional radiology, surgery, and supplemental enteral or parenteral nutrition).

### Exclusion criteria

- Age <18 years old
- Have a length of stay <24 hrs
- Initial diagnosis of BSE is changed or if their readmission is found to be unrelated to previous bariatric surgery.

### Data storage, protection, and validation

Data will be stored on Bedfordshire Hospital NHS Foundation Trust unique NHS.net tenancy. Collaborators need a valid nhs.net or nhs.uk email to access the database using two-factor authentication. Patient data are pseudo-anonymized at the collaborating site before entry. The database will be created using Microsoft PowerApps and Microsoft SharePoint and will predominantly feature drop-down boxes, multiple choice selection, and limited options to ensure uniformity of data recording. Where possible, variable completion will be made compulsory to ensure as close to 100% data completion as possible. There will be a section for free-text typing to allow collaborators. Steering committee members will review the submitted data with a target of >95% case ascertainment.

### Patient identification and data collection

Patient admission lists will be screened daily to identify eligible patients. The data fields will be completed at the earliest opportunity following intervention. Collaborators will actively follow-up patients in the postoperative period.

### Data and variables

Basic demographic data will be captured, which include age, sex, ethnicity, timing of surgery, and ASA. Height and weight will be captured to allow calculation of the body mass index (BMI). Historical weight prior to index bariatric procedure would also be captured to assess weight loss. Data will be recorded on mode of presentation, presenting complaint, diagnosis, and procedures undergone during their admission will be recorded. Data specific to the index bariatric procedure will include the index hospital, the time between index operation and presentation, and previous engagement with NHS obesity pathways.

### Outcome measures

The primary outcomes will include hospital length of stay (general and intensive care wards), 30-day mortality and complications, as per the Clavien–Dindo classification, a standardized, internationally validated scoring system for postoperative complications^[16]^.

Secondary outcomes include the following:

1. National variation in practice and resources utilized in the management of such methods and types of procedures or operations.
2. Rates of outpatient follow-up and further planned procedures (including surgery and endoscopy) following BSE.
3. Requirement for nutritional support, interventional radiology, or admission to critical care units.
4. To determine any potential predictors of participants opting for state-funded versus self-pay bariatric surgery, including ethnicity and previous engagement with NHS-specialized weight loss pathways.

### Statistical analysis

The Shapiro–Wilk test will be used to check variables for Gaussian distribution. Basic demographics will be presented as absolute numbers of participants with the respective percentage per group or as the parameter mean and standard deviation or median and range, depending on distribution. For comparisons of interval-scaled variables, unpaired t tests will be performed. Nonparametric between-group testing will be undertaken with 2-tailed Mann–Whitney U tests. Additionally, the chi-square test or Fisher exact test will be applied to nominal scale data.

Multivariable linear regression be performed for differences among baseline demographics. Analyses will be performed in RStudio with significance defined with *P* < 0.05.

### Authorship

All collaborators returning complete and validated data sets within the timelines will be eligible for collaborative authorship.

### Patient and public involvement

Patient and public involvement (PPI) was established by inviting input from lay members of the NBSR committee who provided guidance on the different phases of the project. Members of the steering committee engaged with people living with obesity separately to ensure their views on the study goals and design were adequately expressed.

The representatives were asked to suggest any important items not already included within the study design and data points and provide feedback on the clarity and acceptability of the study.

### Ethical approval and dissemination

As a clinical audit based on enhancing the outcomes of emergency bariatric surgery, formal ethical approval was not required. Each participating center will register NEBSA locally as a clinical audit. They will also have successfully applied for Caldicott Guardian approval to submit anonymized patient data to a national audit and secure online database. Caldicott approval for Scotland will be secured through a single central application.

The protocol will be disseminated online (tinyurl.com/nesba23), through the BOMMS network and using a social media-based strategy that has been shown to be effective previously (twitter/x: https://twitter.com/NEBSA2023)^[17]^.

## Discussion

The National Emergency Bariatric Surgical Audit (NEBSA) is poised to significantly enhance the current understanding of emergency bariatric surgery practiced across the UK. Preliminary indications suggest that NEBSA will illuminate key aspects of patient demographics, engagement with NHS obesity pathways, and the choice between NHS-funded and self-funded bariatric surgery. By meticulously documenting surgical interventions and outcomes, including length of stay, mortality, admission to critical care, and severity of complications, this study is expected to offer valuable insights into current trends and practices of the management of bariatric complications nationwide. This information could be pivotal in shaping future policy and clinical guidelines to optimize patient care in this rapidly evolving field.

The importance of collaborative efforts in conducting such a comprehensive audit cannot be overstated. The multi-center nature of NEBSA underscores the potential of collaborative research in overcoming the limitations of single-center studies, given the scarcity of bariatric surgical complications. Furthermore, this approach facilitates a broader understanding of variations in practice and resources across different healthcare settings and has been shown to produce high-quality data sets^[17,18]^. By leveraging the collective expertise and data from numerous centres, NEBSA aims to identify best practices and highlight areas experiencing significantly higher rates of intervention. The findings from this audit are expected to catalyze discussions at national forums, thereby informing strategies to bridge knowledge gaps and enhance the overall quality of bariatric surgery care. In particular, collecting information on the nutritional support during treatment of complications arising from bariatric surgery and the need for close monitoring and organ support should further the understanding of the economic burden of this unique type of complications. The National Emergency Bariatric Surgery Audit looks to emulate the success of other national registries such as the National Emergency Laparotomy Audit (NELA), which have driven practice toward incorporating multi-disciplinary treatment^[19]^.

However, it is important to acknowledge the inherent limitations of this study. The main limitation is observational design which does not permit the establishment of causal relationships. While the study can highlight associations and trends in emergency bariatric surgical practices, the findings must be interpreted with caution. The reliance on observational data means that the results might be influenced by unmeasured confounding variables. As a result, the conclusions drawn from this study should be considered as starting points for further research, rather than definitive evidence of causality. Furthermore, information from NEBSA can be juxtaposed against training standards in emergency surgery for bariatric complications, current funding, and workforce planning in providing this specialized care among various specialized medical professionals.

In conclusion, NEBSA represents a significant step forward in our understanding of emergency bariatric surgical practices in the UK. By providing a detailed analysis of current practices, patient outcomes, and various factors influencing these outcomes, this study holds the promise of informing and improving clinical practice and policy-making in the field of bariatric surgery. Being mindful of its limitations, NEBSA’s contributions to the field are expected to be substantial, offering a foundation for future research and a guide for ongoing improvements in patient care.

## Data Availability

Analyzed data can be available post-hoc upon reasonable request.

## Research registration unique identifying number (UIN)

NCT06738004.
